# Detecting and pyramiding target QTL for plant- and grain-related traits *via* chromosomal segment substitution line of rice

**DOI:** 10.3389/fpls.2022.1020847

**Published:** 2022-12-16

**Authors:** Zuyuan Mao, Xinyan Di, Saisai Xia, Qian Chen, Xiaohui Ma, Mei Chen, Zhenglin Yang, Fangming Zhao, Yinghua Ling

**Affiliations:** Chongqing Key Lab of Application and Safety Control of Genetically Modified Crops, Engineering Research Center of South Upland Agriculture, Ministry of Education, Rice Research Institute, Southwest University, Chongqing, China

**Keywords:** rice (*Oryza sativa* L.), chromosome segment substitution lines, grain-related traits, QTL identification, pyramiding

## Abstract

**Introduction:**

Plant height and grain length are important agronomic traits in rice, exhibiting a strong effect on plant architecture and grain quality of rice varieties.

**Methods:**

Methods: A novel rice chromosomal segment substitution line (CSSL), i.e., CSSL-Z1357, with significantly increased plant height (PH) and grain length (GL) was identified from CSSLs constructed by using Nipponbare as a receptor and a restorer line Xihui 18 as a donor. Seven agronomic traits of PH, PL, GL, GW, GPP, SPP, and TGW were phenotyped, and REML implemented in HPMIXED of SAS were used to detect the QTL for these traits. Secondary CSSLs were screened out via marker-assisted selection (MAS) to estimate the additive and epistatic effects of detected QTLs, evaluating the potential utilization of pyramiding the target QTLs for yield and quality improvement of rice varieties.

**Results and Discussion:**

Results and Discussion: CSSL-Z1357 carried nine segments from Xihui 18 with an average segment length of 4.13 Mb. The results show that the long grain of CSSL-Z1357 was caused by the increased number of surface cells and the length of the inner glume. Thirteen quantitative trait loci were identified via the F2 population of Nipponbare/CSSL-Z1357, including three each for GL (qGL-3, qGL-6, and qGL-7) and PH (qPH-1, qPH-7, and qPH-12I), among which qGL-3 increased GL by 0.23 mm with synergistic allele from CSSL-Z1357. Additionally, three single (S1 to S3), two double (D1, D2), and one triple segment (T1) substitution lines were developed in F3 via MAS. Results show that pyramiding the segments from Chr.3 (qGL-3 and qPH-3), Chr.6 (qGL-6 and qPH-6), and Chr.7 (Null and qPH-7) tended to result in better phenotype of increased GL and PH and decreased grain width, providing a potential basis for enhancing grain yield and quality in rice breeding.

## Introduction

Grain-related traits, including length, width, thickness, and weight, in rice (*Oryza sativa* L.) determine yield and quality of rice varieties, making them important for both breeders and scientists (Xu et al., 2004). Up to now, a large number of major quantitative trait loci (QTLs) controlling grain length (GL) and other related traits were detected; some of these QTLs were cloned and functionally profiled, e.g., *BG1*, *PGL1*, *GL2/3*, *GS2/3/9*, *GSE5*, and *GW2/5/7*. In addition, several signaling pathways, including phytohormones, ubiquitin-proteasome, G-protein, mitogen-activated protein kinase (MAPK), transcription factors (TFs), and secreted peptides, that control grain size have been revealed (Fan and Li, 2019; Li et al., 2021). Brassinosteroids (BRs), auxin, and cytokinin (CK) are the phytohormones that play important roles in controlling grain size (Che et al., 2015).

In rice, more than 10 genes are reported to regulate grain size through phytohormone-related pathways. For example, eight genes, i.e., *GSE5*/*GW5* (Weng et al., 2008; Duan et al., 2017; Liu et al., 2017), *GSK2* (Tong et al., 2012; Lyu et al., 2020), *GSK3* (Gao et al., 2019; Liu et al., 2021), *GS5* (Li et al., 2011), *GS2* (Duan et al., 2015; Hu et al., 2015), and *qGL3*/*OsPPKL1* (Zhang et al., 2012; Gao et al., 2019), are documented to regulate rice grain size *via* the BR signaling pathway. In addition, six genes [*qTGW3* (Hu et al., 2018), *BG1* (Liu et al., 2015), *TGW6* (Ishimaru et al., 2013), *RBG1* (Lo et al., 2020), and *qGL5/OsAUX3* (Qiao et al., 2021)] associated with the auxin pathway, and two genes [*OsPUP7* (Qi and Xiong, 2013) and *BG3* (Xiao et al., 2019; Yin et al., 2020)] linked to the CK pathway are reported to regulate grain size in rice.

The ubiquitination-proteasome pathway genes, including *GW2* (Song et al., 2007; Hao et al., 2021), *OsOTUB1*/*WTG1* (Wang et al., 2017; Huang et al., 2017), *LG1*/*OsUBP15* (Shi et al., 2019), and *TUD1*(Hu et al., 2013), also play crucial roles in the regulation of grain size. The genes *DEP1*, *GGC2*, and *GS3* (Fan et al., 2006; Mao et al., 2010; Sun et al., 2018; Liu et al., 2018); *D1/RGA1*, *RGB1* (Zhang et al., 2021a); *RGG1* (Tao et al., 2020); *RGG2* (Miao et al., 2019); and *LGY3* (Liu et al., 2018) belonging to the G-protein pathway are also involved in regulating grain size (Liu et al., 2018; Sun et al., 2018). In addition, the combined MAPK module of OsMKKK10-OsMKK4-OsMAPK6 positively regulates grain size by affecting cell proliferation in rice (Xu et al., 2018; Fan and Li, 2019; Li et al., 2021). The MAPK pathway can promote rice grain development *via* the activation of *OsWRKY53*, whereas *OsMKP1*/*GSN1* tends to suppress *OsMAPK6* and, thus, depress the functioning of this pathway (Fan and Li, 2019; Li et al., 2021). Several TFs are also involved in the regulation of grain size in rice, such as *GLW7*/*OsSPL13* (Si et al., 2016), *OsSPL16*/*GW8* (Wang et al., 2012), *GS9* (Zhao et al., 2018), *An-1* (Luo et al., 2013), and *GL6*/*SG6* (Wang et al., 2019; Zhou and Xue, 2020).

*GS5* is a major QTL controlling grain width (GW), grain filling, and grain weight in rice (Li et al., 2011). The product of *GS5*, a putative serine carboxypeptidase, tended to regulate the grain size positively, thus serving as a potential candidate for yield improvement in rice and other cereals (Li et al., 2011). The encoded protein of *GS9* had a conserved domain with unknown function that could alter cell division and regulate grain shape in rice (Zhao et al., 2018). In addition to regulating grain shape, the *GS9* allele exhibited functions of improving grain appearance (as a marker of quality) in rice (Zhao et al., 2018).

Some other phenotypic traits also contribute to the grain size in rice. For example, grain number per panicle/plant, a typical quantitative trait that determines rice yield, generally correlates negatively with grains size traits (Lu et al., 2022). Downregulation of *GW10* that encodes a P450 subfamily protein in rice tends to result in decreased GL and GW but increased grain number (Zhan et al., 2021). A loss of function of *gad1* (*grain number, grain length and awn development1*) caused by a frame-shift insertion increases grain number while decreasing GL in rice (Jin et al., 2016). Plant height (PH) is another typical quantitative trait that determines rice plant architecture and affects the yield of rice varieties. Although weak correlations between PH and GL are documented in rice (Sabesan et al., 2009; Lakshmi et al., 2014), some genes for PH have pleiotropic effects influencing GL in rice. Huang and colleagues reported that the rice mutant *ZPDM1* with a new allele of *BRD2* (*BRASSINOSTEROID DEFICIENT DWARF 2*) tended to result in the phenotype of reduced PH and smaller grain size (Huang et al., 2022). On the other hand, a novel rice germplasm with increased grain size but decreased height was developed, confirming the weak correlation between PH and GL or size. Tomita and colleagues pyramided grain size–related gene *GW2* and plant height-related gene *sd1* in the background of Koshihikari in rice, and the newly developed line Koshihikary-*sd1GW2* exhibited significantly increased GL and GW but semi-dwarfed PH (Tomita et al., 2022).

The profiling of major QTLs for grain-related traits enriches our knowledge of grain architecture in rice as well as provides potential genes for molecular breeding in rice variety improvement. However, grain-related traits exhibit typical quantitative characteristics with incompletely dissected molecular mechanisms of inheritance. Hence, new novel grain-related mutants are needed. In our previous work, we introduced chromosomal segments from Xihui 18 rice, an elite restorer line, to the background of Nipponbare, constructing a series of chromosome segment substitution lines (CSSLs). From these CSSLs, we identified a distinctive CSSL-Z1357 with significantly increased GL, using SSR-based marker-assisted selection (MAS). In the present study, we characterized CSSL-Z1357, constructed a segregating population to detect QTLs for GL and other traits of interest, and estimated their effects under independent and pyramiding backgrounds. Related results provide a basis for candidate gene dissection and grain-related trait improvement in rice breeding.

## Materials and methods

### Materials and population construction

CSSL-Z1357 (Z1357) was screened out from the CSSLs produced from the crossing of Nipponbare (receptor) and Xihui 18 (donor). Z1357 carried nine chromosomal segments from the donor Xihui 18 as asserted *via* SSR-based MAS. The F2 population was constructed by crossing Z1357 and the receptor Nipponbare for QTL mapping of agronomic traits of interest. In 2017, F2 individuals containing target QTLs were self-pollinated to construct advanced CSSLs, including three lines carrying a single substitution segment (SSSL, S1 to S3), two lines with double substitution segments (DSSL, D1 and D2), and one line with triple-substitution segments (TSSL, T1). In 2020, all six screened lines (S1 to S3, D1, D2, and T1) were used to estimate the additive and epistatic effects of target QTLs.

### Phenotyping of specific agronomic traits

At maturity, more than eight randomly selected plants of Z1357 and Nipponbare and all F2 individuals were cut at the ground level; then plant height (PH, cm) and panicle length (PL, cm) were measured. After air-drying, the grain number (filled grains) and spikelet number (total grains of both filled and unfilled grains) per panicle (abbreviated as GPP and SPP, respectively) were counted; 1000 filled grains were randomly selected for measuring of 1000-grain weight (TGW, g). Ten random filled grains were selected for measuring grain length (average grain length of 10 grains, GL, mm) and grain width (average grain width of 10 grais, GW, mm).

### Observation of epidermal cells *via* scanning electron microscope

During the early heading stage, young panicles of Z1357 and Nipponbare were harvested and placed on ice for scanning electron microscope (SEM) examination. About 5 mm segments of middle glume were excised for observation of inner and outer epidermal cells according to the procedures described by Zhuang et al. (2020). The cell length and cell number of the inner and outer epidermis and outer parenchyma of glume within the same field of view were collected *via* software Simpal PCI.

### DNA extraction and molecular mapping

DNA of all lines was extracted *via* the CTAB procedure. All the F2 individuals were genotyped *via* the polymorphic markers screened by the introgressed segments of Z1357 (Xiang et al., 2015). QTLs for seven agronomic traits of interest, i.e., PH, PL, GPP, SPP, TGW, GL, and GW, were detected by the method of restricted maximum likelihood (REML) implemented in HPMIXED of SAS. The threshold for identifying the association of candidate QTL and the particular trait was set to *P* <.05. All detected QTLs were named as per the pattern of *qPH-1*, whereby *q* referred to a QTL; *PH* to the target trait, i.e., PH; and the number to the chromosome of QTL location.

### Effect estimating of QTL *via* advanced CSSLs

For each SSSL, DSSL, and TSSL, according to the genetic model *P_0_* = *μ* + *ϵ* for Nipponbare and *P_i_* = *μ* + *a_i_* + *ϵ* for the SSSL*_i_* carrying a specific QTL, *P_ij_* = *μ* + *a_i_* + *a_j_* + *I_ij_* + *ϵ* for DSSL, and *P_ijk_* = *μ* + *a_i_* + *a_j_* + *a_k_* + *I_ijk_* + *ϵ* for TSSL, *P_0_* and *P_i_* represent the phenotype value of any plant of Nipponbare and the SSSL*_i_* carrying the substitution segment *i*; *P_ij_* and *P_ijk_* represent the phenotype value of any plant in the DSSL*_ij_* and TSSL*_ijk_*; *μ* represents the mean value of Nipponbare population; *a_i_*, *a_j_*, and *a_k_* represent the additive effect of the QTL in substitution segments *i*, *j*, and *k*, respectively; *I_ij_* and *I_ijk_* represent the *a_i_a_j_* epistatic effect between QTLs in substitution segments *i* and *j*; *a_i_a_j_a_k_* denotes the epistatic effect between QTLs in the substitution segments *i*, *j*, and *k*, respectively; and *ϵ* represents the random error (Liang et al., 2021; Wang et al., 2021). Thus, the additive effect to the target trait was estimated *via* the formula (SSSL_i_-Nipponbare)/2 based on S1 to S3. The epistatic effect of each QTL pair (or substitution segment pair) to the control trait was estimated *via* the formula ((Nipponbare + DSSL*_ij_*) – (SSSL*_i_* + SSSL*_j_*))/2 based on both DSSLs (D1 and 2) and SSSLs (S1 to S3). The epistatic effect of three QTLs (or three substitution segments) to the control trait was estimated *via* ((Nipponbare×2+TSSL*_ijk_*) – (SSSL*_i_* +SSSL*_j_* +SSSL*_k_*))/2 (Liang et al., 2021; Wang et al., 2021). All calculations were carried out using Microsoft-Excel 2016.

## Results

### Phenotypic characterization of Z1357

**Figure 1:**
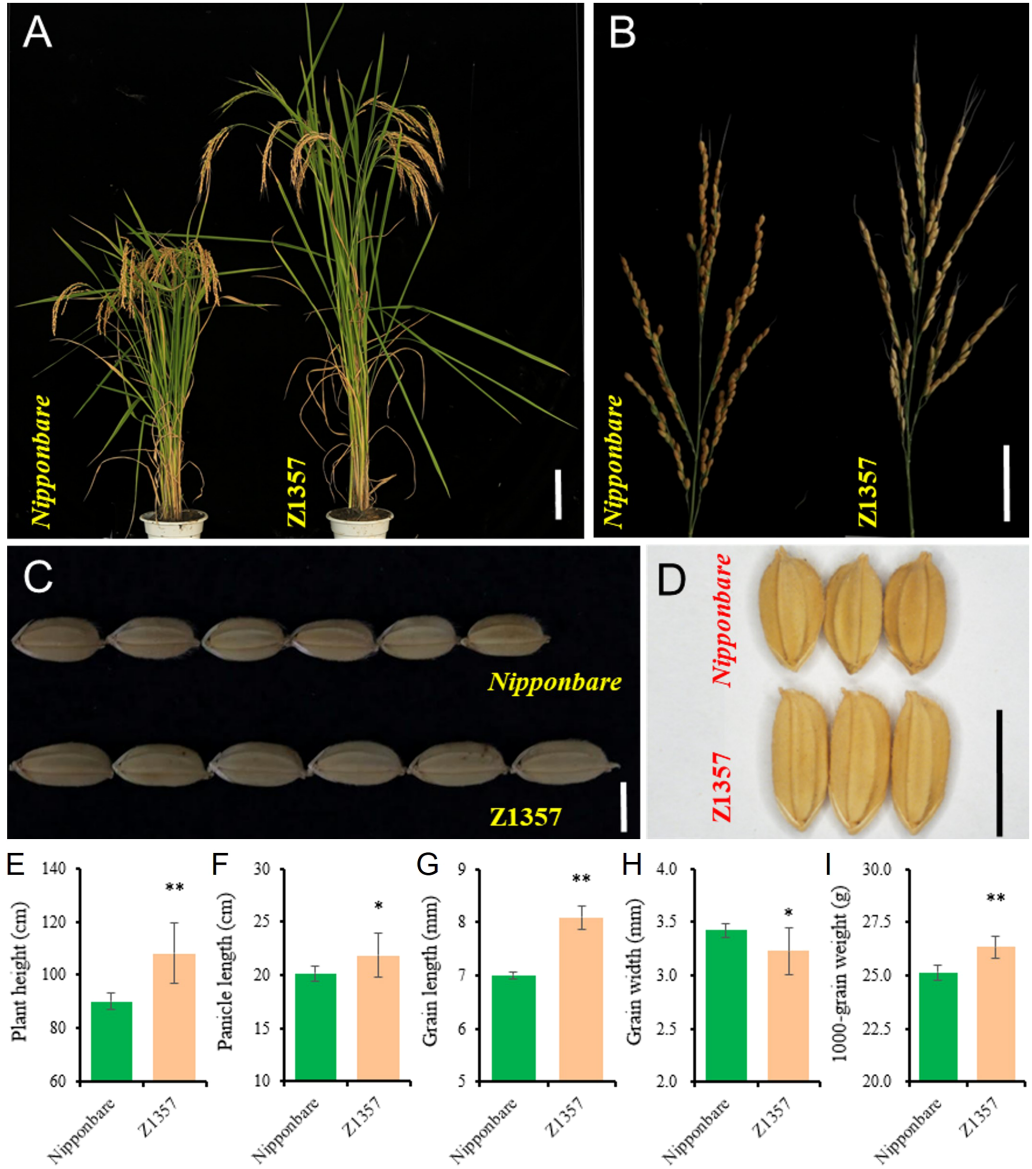
Phenotypes of Nipponbare and Z1357. **(A)** Plant architecture of Nipponbare and Z1357; **(B)** Panicles of Nipponbare and Z1357; **(C, D)** Phenotypes of grain length **(C)** and width **(D)** of Nipponbare and Z1357; **(E–I)** Comparison of PH **(E)**, PL **(F)**, GL **(G)**, GW **(H)**, and TGW **(I)** between *Nipponbare* and Z1357. Bar in A refers to 20 cm, in B to 5 cm, in C and D to 1 cm. * and ** refer to the significant differences at P < .05 and P < .01, respectively, by the t-test.

Phenotyping results show that Z1357 exhibited significantly changed plant and panicle/grain architecture compared with the receptor, i.e., Nipponbare (Nipp) (**Figures 1A–D**). At maturity, seven traits of interest, including PH (cm), PL (cm), SPP, GPP, GL (mm), GW (mm), and TGW (g), were further characterized between Z1357 and Nipp. Both Z1357 and Nipp had similar SPP (104.90 ± 2.96 for Z1347 and 105.10 ± 9.66 for Nipp) and GPP (94.96 ± 5.07 for Z1347 and 98.99 ± 9.40 for Nipp), exhibiting nonsignificant differences (*P* >.05). The contrasting trends were observed for the remaining five traits (**Figures 1E–I**). Z1357 had statistically higher PH, PL, GL, and TGW than those of Nipp (**Figures 1E–I**). However, the average GW of Z1357 was 3.23 mm, significantly lower than that of Nipp (3.42 mm, *P* <.05**, Figure 1H**).

### Cytological analysis of Z1357

**Figure 2:**
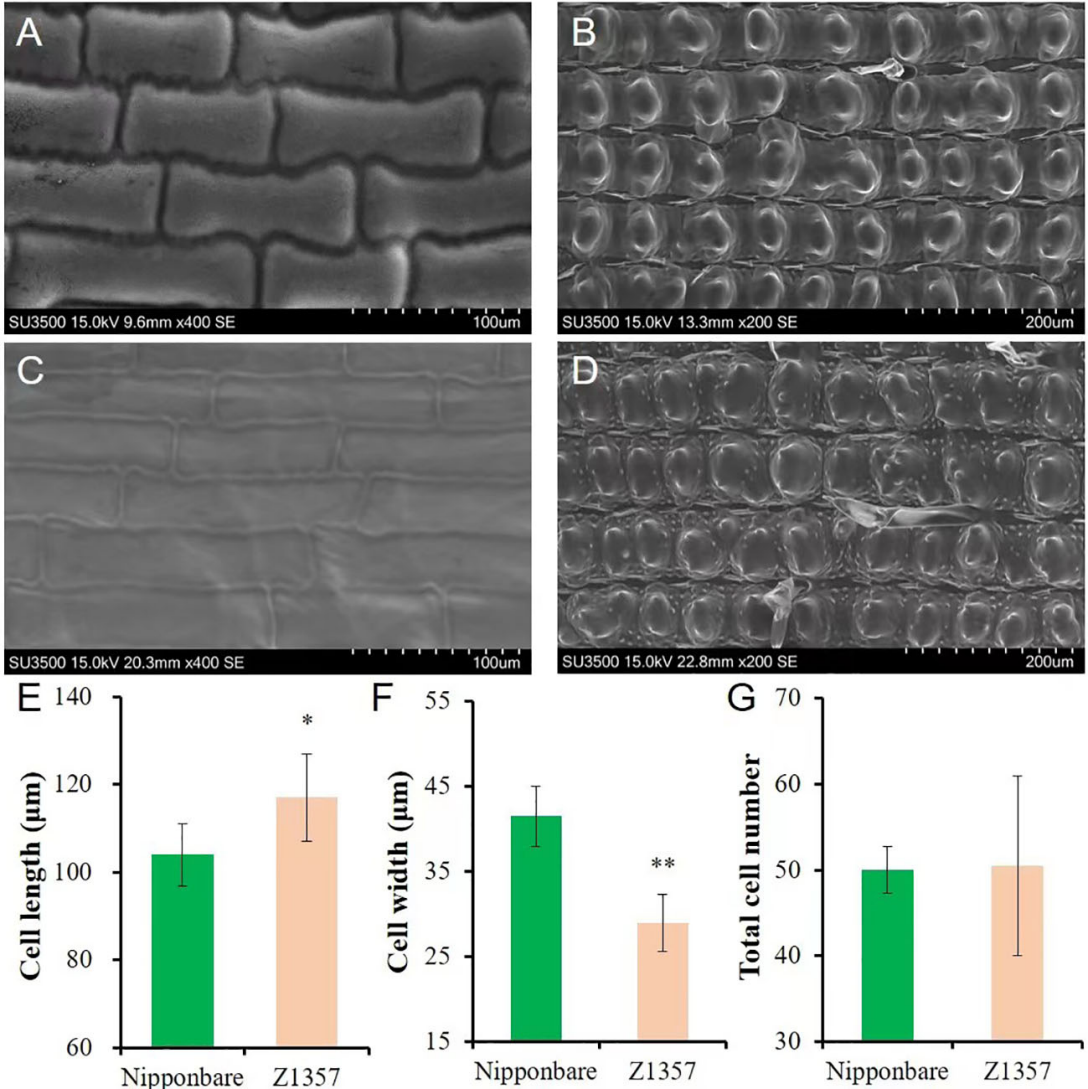
Observation and analysis of glume cells between *Nipponbare* and Z1357 *via* scanning electron microscope (SEM). A-B: SEM images of glume inner **(A)** and surface **(B)** cells of *Nipponbare*; **(C, D)** SEM images of glume inner **(C)** and surface **(D)** cells of Z1357; E-F: Comparison of inner glume cell length **(E)** and width **(F)** between *Nipponbare* and Z1357; **(G)** Comparison of glume surface cells number between *Nipponbare* and Z1357. * and ** refer to the significant differences at *P* < .05 and *P* < .01, respectively, by the *t*-test.

The morphology of glume cells was observed by SEM (**Figures 2A–D**). The results show that the length of glume inner cells of Z1357 was significantly longer (12.48%) than that of Nipp (**Figures 2A, C**) (*P* <.05, *t*-test, **Figure 2E**). On the other hand, the cell width of Z1357 glumes (33.55 µm) was statistically smaller than that of Nipp (40.86 µm) (*P* <.01, **Figure 2F**), but no significant difference was observed between cell numbers of Z1357 and Nipp (**Figure 2G**). These results suggest that the increased GL of Z1357 was mainly caused by the increased length of glume cells.

### Molecular characterization of Z1357

**Figure 3:**
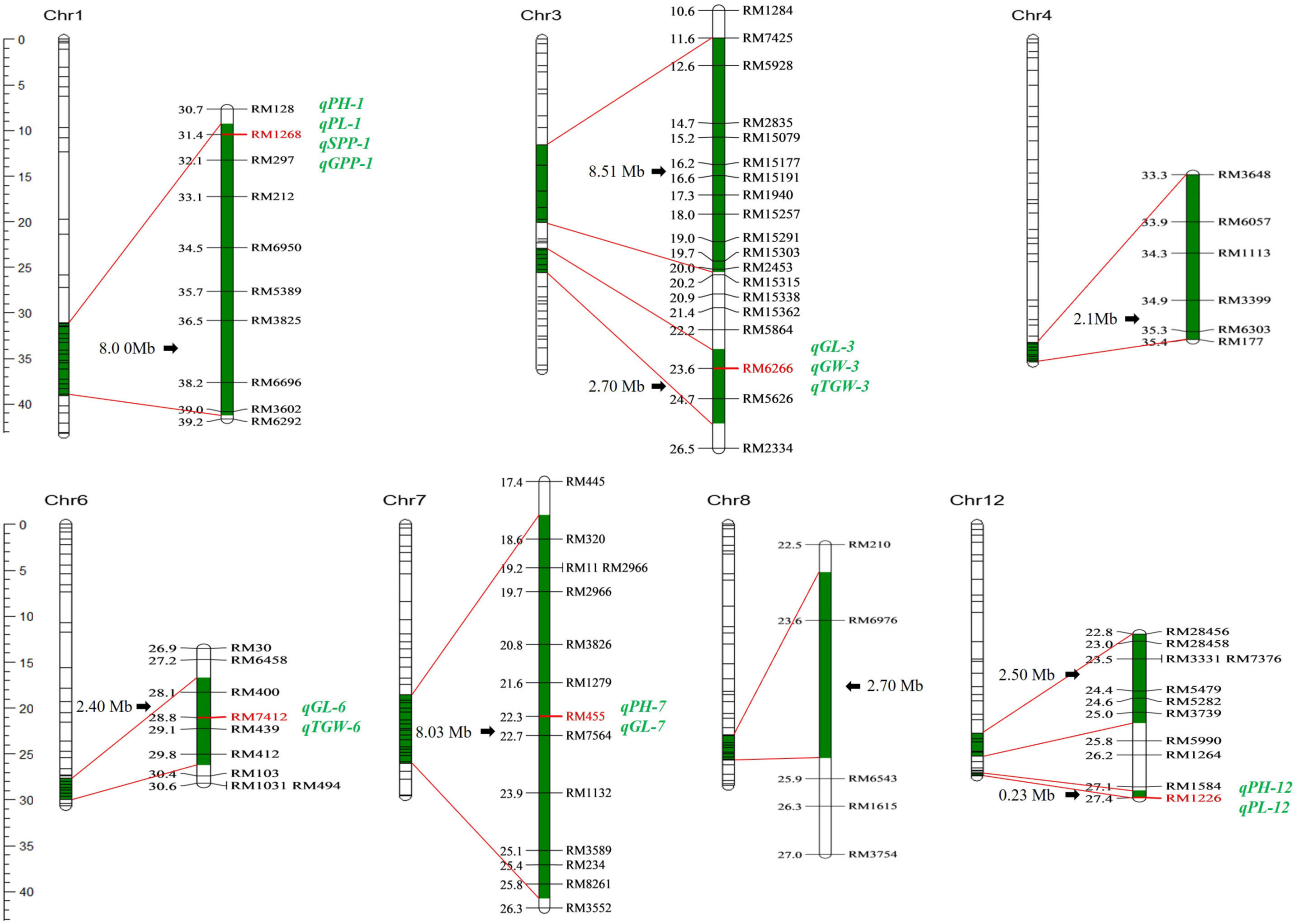
Genome distribution of substitution segments in Z1357. Substitution fragments are labeled in dark green. The left bar refers to the physical position (Mb) of markers (short lines of each Chr. bar) on seven chromosomes. All detected QTLs are listed in italics (green), and the linked marker of each QTL is listed in bold (red).

Nine segments were introduced into the genome of Z1357, located on seven chromosomes (Chr): Chr1, Chr3, Chr4, Chr6 to Chr8, and Chr12. The characterization results of 10 selected molecular markers showed that the introgressed segments carried by Z1357 exhibited the same genotypes to those of the donor line, Xihui18, indicating that the introgressed chromosomal segments from Xihui18 were homologous in the genome of Z1357. Among all seven Chrs carrying the introgressed segments, Chr3 and Chr12 contained two introgressed segments each, and the remaining five chromes had only one segment each (**Figure 3**).

The total estimated length of all the nine introgressed segments was 37.17 Mb with an average of 4.13 Mb. The largest segment was detected on Chr3 with a length of 8.51 Mb (**Figure 3**). The shortest segment was detected on Chr12 with a length of 0.23 Mb (**Figure 3**). The length of the remaining seven segments ranged from 2.1 Mb to 8.03 Mb.

### QTL mapping of the traits of interest

A total of 13 QTLs were detected for six agronomic traits (**Table 1**; **Figure 3**). These QTLs were located on five chromosomes, including Chr1 (four QTLs), Chr3 (three QTLs), Chr6 (two QTLs), Chr7 (two QTLs), and Chr12 (two QTLs) (**Table 1**; **Figure 3**). No QTLs were detected for any traits of interest on the first introgressed segment in Chr3 (from the top), nor introduced segments in Chr4 and Chr8 (**Table 1**; **Figure 3**).

**Table 1:** Summary of 13 QTLs identified for the six interest traits *via* the F2 population of Nipponbare/Z1357.

Traits	QTL	Chr.	Linked marker	Estimated effect	Var (%)	P-value
Plant height (PH, cm)	*qPH-1*	1	RM1268	-3.38	4.76	.0004
	*qPH-7*	7	RM455	2.12	1.75	.0282
	*qPH-12*	12	RM1226	2.67	3.37	.0040
Panicle length (PL, cm)	*qPL-1*	1	RM1268	-0.37	1.95	.0196
	*qPL-12*	12	RM1226	0.49	3.80	.0018
Grain length (GL, mm)	*qGL-3*	3	RM6266	0.23	36.49	<.0001
	*qGL-6*	6	RM7412	0.08	4.19	.0065
	*qGL-7*	7	RM455	0.12	8.84	<.0001
Grain width (GW, mm)	*qGW-3*	3	RM6266	-0.03	3.27	.0011
1000-grain weight (TGW, g)	*qTGW-3*	3	RM6266	0.57	6.65	<.0001
	*qTGW-6*	6	RM7412	0.54	5.65	.0009
Grain number per panicle (GPP)	*qGPP-1*	1	RM1268	-7.05	5.89	<.0001
Spikelet number per panicle (SPP)	*qSPP-1*	1	RM1268	-6.25	3.63	.0008

Three QTLs, i.e., *qPH-1*, *qPH-7*, and *qPH-12*, were detected for PH and were located on Chr1, Chr7, and Chr12. Mapping results showed that *qPH-1* could decrease PH by 3.38 cm, whereas *qPH-7* and *qPH-12* increased it by 2.12 and 2.67 cm, respectively, with Var <5% (**Table 1**). Two QTLs controlling PL, *qPL-1* and *qPL-12*, were located on Chr1 and Chr12 (**Table 1**; **Figure 3**). Both *qPH-1* and *qPL-1* shared the same linking marker (RM1268, **Figure 3**).

Three QTLs, i.e., *qGL-3*, *qGL-6*, and *qGL-7*, were detected for GL and were located on Chr3, Chr6, and Chr7 (**Figure 3**). Mapping results showed that *qGL-3*/*6*/*7* was associated with the additive effects of 0.23, 0.08, and 0.12 mm to GL with Var of 36.49%, 4.19%, and 8.84%, respectively (**Table 1**). The linking marker of *qGL-3* was RM6266 and the two other QTLs, i.e., *qGW-3* and *qkGW-3*, controlling GW and TGW, respectively, also linked with the same marker (**Table 1**; **Figure 3**). Additionally, *qTGW-3* exhibited relatively higher Var (6.65%) regarding TGW (**Table 1**).

Only one QTL was detected for both SPP and GPP, i.e., *qSPP-1* and *qGPP-1* (**Figure 3**). They shared the same linking marker (RM1268) with *qPH-1* and *qPL-1*, with the corresponding Var values of 3.63% and 5.89% (**Table 1**).

### Verification and estimation of additive effects of target QTLs

**Figure 4:**
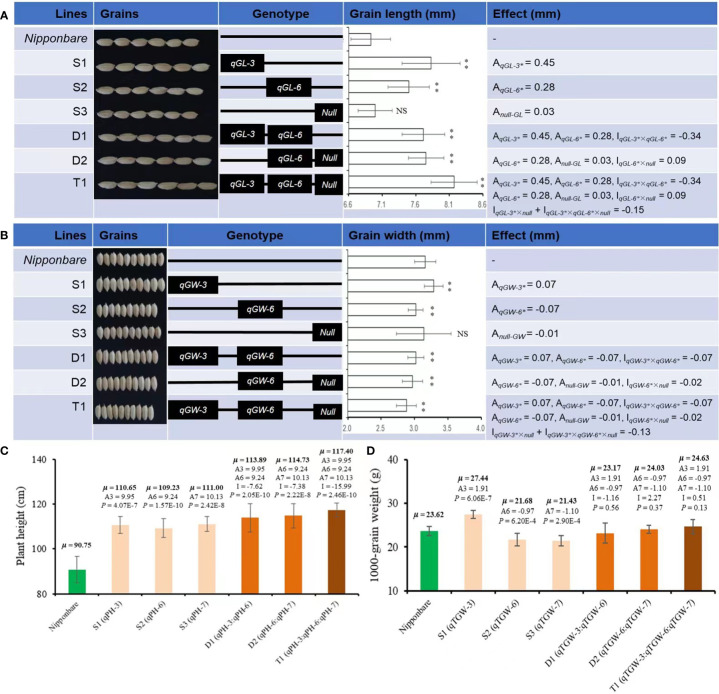
Phenotypes and estimated effects of QTLs for GL, GW, PH, and TGW among Nipponbare and the screened CSSLs. **(A)** Grain length (mm), **(B)** Grain width (mm), **(C)** Plant height (cm), **(D)** 1000-grain weight (g). μ: the average phenotypic value, a: denotes the additive effect of QTLs, i: denotes the additive × additive epistatic effect among QTLs. **, significant at.01 level (*P* < .01); NS, not significant. The *P*-value for a SSSL indicates the probability of a significant difference between the SSSL and Nipponbare.

Based on the QTL mapping results using F2, three SSSLs (S1, S2, S3) were screened out from the F3 generation *via* MAS. Among these SSSLs, S1 carried four QTLs on Chr3 for GL, GW, PH, and TGW, and they had positive additive effects of 0.45 and 0.07 mm, 9.95 cm, and 1.91 g, respectively, causing statistically higher values of these traits than those of the receptor genotype Nipp (**Figure 4**). The line S2 also carried four QTLs for GL, GW, PH, and TGW. These QTLs were on Chr6. Two QTL (for GL and PH) showed positive additive effects of 0.28 mm and 9.24 cm, respectively, whereas the other two QTLs (for GW and TGW) exhibited negatively additive effects of -0.07 mm and -0.97 g, respectively, causing significantly increased GL and PH but significantly decreased GW and TGW than those of Nipp (**Figure 4**). The line S3 contained two QTLs for PH and TGW, presenting additive effects of 10.13 cm and -1.10 g, respectively, causing statistically increased PH but deceased TGW compared with those of Nipp (**Figure 4**).

### Pyramiding and estimation of epistatic effects of target QTLs

Besides SSSLs (S1-S3), two DSSLs (D1 and D2) and one TSSL (T1) were also purposefully screened out from F3 generation *via* MAS according to the target QTL. D1 carried two chromosomal segments from Chr3 and Chr6 for GL, GW, PH, and TGW, and the broad interactive or epistatic effects (I) were detected for QTLs controlling the corresponding traits (**Figure 4**). For GL, the estimated I between *qGL-3* and *qGL-6* was -0.34 mm, and the pyramiding of these two QTLs caused significantly greater GL of D1 than that of Nipp (**Figure 4A**). A contrasting trend was observed for GW of D1. The estimated I between *qGW-3* and *qGW-6* was -0.07 mm, and the pyramiding of these two QTLs caused significantly decreased GW of D1 compared with Nipp (**Figure 4B**). Additionally, the estimated I between *qPH-3* and *qPH-6* was -7.62 cm. The epistatic effect between *qTGW-3* and *qTGW-6* was -1.16 g for TGW; the interaction effects caused significantly increased PH but nonsignificant changed TGW in D1 compared with Nipp (**Figures 4C, D**).

D2 captured two segments from Chr6 and Chr7. Although D2 carried only one QTL for each of GL (*qGL-6*) and GW (*qGW-6*), the interaction effects (I) were also observed between the introgressed segments with and without (*Null*) the target QTL. The estimated I between *qGL-6* and *Null* was 0.09 mm, whereas the estimated epistatic effect between *qGW-6* and *Null* was -0.02 mm (**Figures 4A, B**). These interaction effects caused significantly increased GL but decreased GW of D2 (*P* <.01), which was similar to the findings on D1 (**Figures 4A, B**). The estimated difference between *qPH-6* and *qPH-7* was -7.38 cm, and that between *qTGW-6* and *qTGW-7* was 2.27 g (**Figures 4C, D**). These diverse interaction effects for PH and TGW caused similar phenotypes of D2 and D1 by the pyramiding of the target QTL.

**Figure 5:**
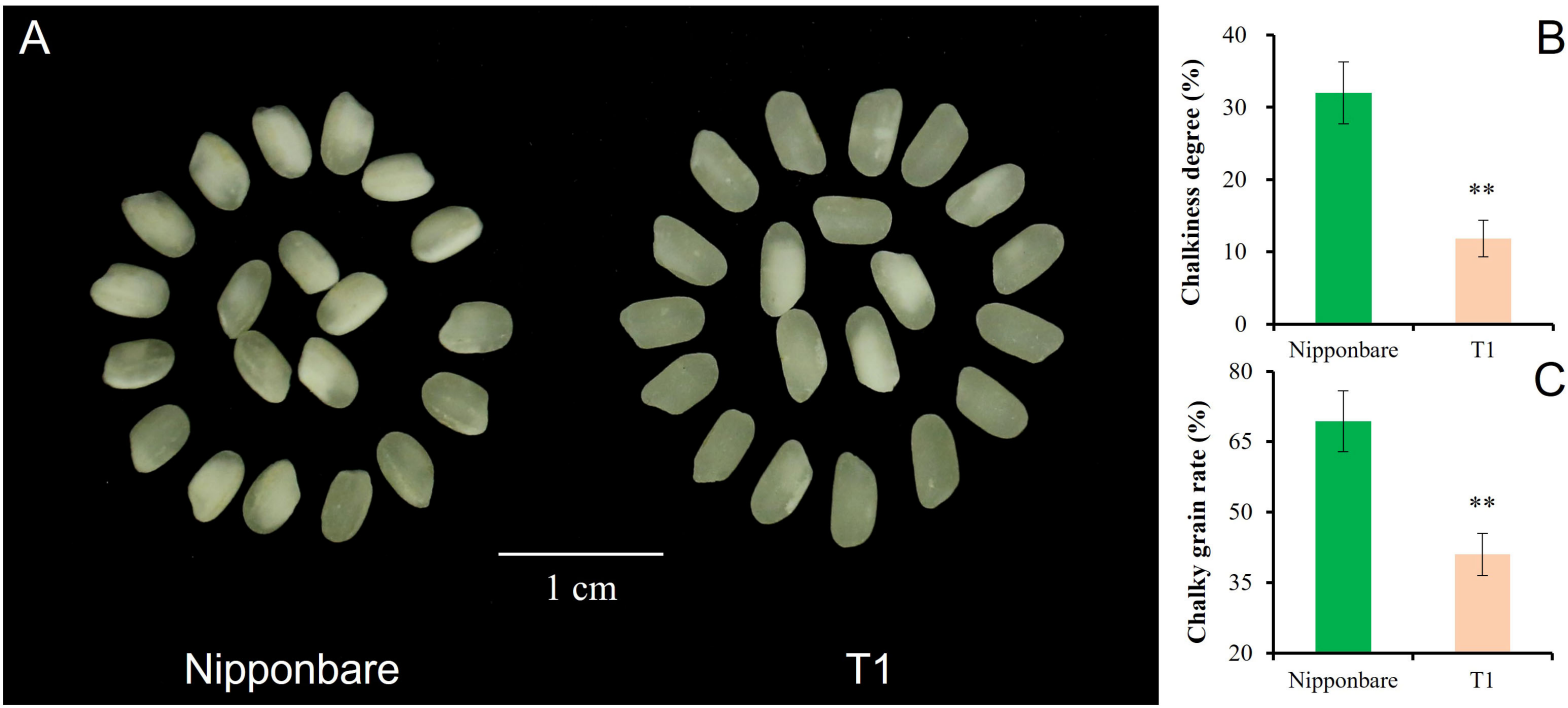
Chalkiness comparison of Nipponbare and T1. **(A)** Polished grain of Nipponbare and T1; **(B)** Chalkiness degree (%) of Nipponbare and T1; **(C)** Chalky grain rate (%) of Nipponbare and T1. **, significant at.01 level (*P* < .01).

T1 captured all three segments from Chr3, Chr6, and Chr7. The pyramiding of *qGL-3*, *qGL-6*, and *Null* caused the highest GL of T1, and the estimated interaction effect of *qGL-3* vs *Null* and *qGL-3* vs *qGL-6* vs *Null* was -0.15 mm (**Figure 4A**). In contrast to GL, T1 also had the lowest GW by pyramiding *qGW-3*, *qGW-6*, and *Null*, and the estimated interaction effect of *qGW-3* vs *Null* and *qGW-3* vs *qGW-6* vs *Null* was -0.13 mm (**Figure 4B**). Trends similar to those of GL were also observed for PH and TGW of T1. The estimated interaction effect of *qPH-3* vs *qPH-6* vs *qPG-7* was -15.99 cm, and that of *qTGW-3* vs *qTGW-6* vs *qTGW-7* was 0.51 g; hence, pyramiding of these QTLs significantly increased PH (*P <*0.01) but did not significantly change TGW (*P <*0.13) of T1 (**Figures 4C, D**). Additionally, the pyramiding of targets QTLs in T1 also caused improved quality of T1 grains (**Figure 5A**). For example, the chalkiness rate of T1 grains was 11.86%, statistically lower than that of Nipp (32.00%, *P* <.01, **Figure 5B**). The overall chalky grain rate of T1 (41.02%) was also significantly lower than that of Nipp (69.36%, *P* <.01, **Figure 5C**).

## Discussion

### Z1357 provides a potential tool for dissecting quantitative traits

Populations consisting of SSSLs are one of the major sources for QTL detection of complex quantitative traits for the distinctive features of eliminating background influences to the mapping procedures (Li et al., 2005; Tian et al., 2017; Chen et al., 2018; Zhang, 2021b). SSSLs have a background nearly the same as the receptor parent except for the introduced segment from the donor (Li et al., 2005; Chen et al., 2018; Tian et al., 2018). In our previous work, a set of CSSLs were developed by crossing Nipponbare (donor parent) and Xihui 18 (donor parent), and a distinctive line, Z1357, was screened out with significantly increased GL and PH but decreased GW (**Figure 1**). Results from phenotypic characterization also suggest that the introduced chromosomal segments from donor parent into Z1357 showed similar traits of GPP and SPP to those of the receptor parent (*P* >.05, **Figure 1**). Although the final yield of Z1357 was significantly lower than that of the receptor, the distinctive characteristics of Z1357 regarding increased GL (15.6%, *P* <.01) and PH (20%, *P* <.01), but decreased GW (5.6%, *P* <.05) showed a potential for utilization in grain quality improvement in rice breeding and research.

SEM showed that the lengthening and narrowing of grains were caused by the increase of cell length (12.48%) and the decrease of cell width (17.89%) in the glumes compared with Nipponbare (**Figure 2**). Dissection of other traits of interest using Z1357 would be useful in the related future research on these traits.

### Identification of QTLs *via* Z1357 and comparison with the reported genes

The QTL mapping results based on the F2 indicated that five out of nine substitution segments contained in Z1357 carried QTLs for six traits of interest, i.e., QTLs for PH and PL on segments of Chr1, Chr7, and Chr12 and QTLs for GL and GW on Chr3 and Chr6, respectively (**Table 1**). We also found that each introduced fragment contained multiple QTLs for different traits, and for each trait, the detected QTLs were located on different introgression segments, which indicated a complex cross-talk between introgression segments and the traits of interest.

Grain type in rice, especially GL and GW, is one of the most important components for quality improvement of rice varieties. As expected, GL was a typical quantitative trait and was controlled by both the major and minor QTLs. Numerous QTLs associated with GL have been reported across the entire rice genome using populations of F2, F3, and recombinant inbred lines (Zhang et al., 2021). However, the features of those populations limit the thorough dissection of candidate QTLs, such as cloning and functional profiling. In the present study, we identified the major QTLs for GL and GW within the introduced segments of Chr3 and Chr6, respectively; in particular, *qGL-3* on Chr3 increased GL by 0.23 mm with Var of 36.5% (**Table 1**). At the locus of *qGL-3*, a functional gene of *GL3.1* controlling the GL in rice was reported (Qi et al., 2012). The coding product of *GL3.1* is Ser/Thr phosphatase that belongs to protein phosphatase kelch-like (PPKL) family (Qi et al., 2012). Qi and colleagues revealed that *GL3.1* functioned by influencing the phenotype of grain size and yield of rice *via* regulating a cell cycle-related protein (cyclin-T1;3) *via* dephosphorylation (Qi et al., 2012). The downregulation of cyclin-T1;3 by dephosphorylation tended to produce shortened rice grains (Qi et al., 2012).

On Chr6, the reported functional gene, *GW6a*, was located in the same region as *qGL-6* (Song et al., 2015). The product of *GW6a* is a new-type GNAT-like protein, serving as intrinsic histone acetyltransferase (OsglHAT1) (Song et al., 2015). Overexpression of *OsglHAT1* tended to increase the overall acetylation levels of histone H4 and the cell number in grains, resulting in enlarged grain size and enhanced final yield of rice (Song et al., 2015). Another reported gene, *GW7/GL7* is located in the same region as *qGL-7* (Wang et al., 2015a, b). The product of *GW7/GL7* corresponds to longitudinal cell elongation, homologous to the LONGIFOLIA protein of *Arabidopsis* (Wang et al., 2015). The increased abundance of *GW7/GL7* coding product *in vitro* accelerated the longitudinal cell division and decreased the transverse cell division, resulting in increased GL and improved grain quality in terms of appearance (Wang et al., 2015).

The gene *OsBZR1* is responsible for PH in rice and is located in the physical interval of *qGL-7* (Qiao et al., 2017). The *OsBZR1* serves as the signal molecule downstream of the brassinolide transduction pathway (Qiao et al., 2017). Overexpression of *OsBZR1* tended to increase the sugar accumulation in developing anthers and seeds and also enhanced GL, GW, thickness, TGW, and spikelet number (Qiao et al., 2017). In summary, the QTLs reported in the present study (*qGL-3*, *qGL-6*, and *qGL-7*) were located within the same regions as the four genes reported in the literature, i.e., *GL3.1*, *GW6.1*, *GW7/GL7*, and *OsBZR1*. Further work is needed to reveal whether those three QTLs contain the allelic variations of the reported genes.

### Potential utilization of the identified QTLs in rice grain improvement

Based on the results of QTL mapping, six further substitution fragment lines (S1-S3, D1-D2, T1) were screened out (**Figure 4**). Characterization of these six lines provides the practical knowledge for molecular or QTL-based improvement of target traits in rice. For example, in rice variety improvement, breeders prefer to select longer grains or grains with higher ratio of length/width (Li et al., 2021). The results of mapping and effect estimation indicated that *qGL-3* and *qGL-6* could both increase the GL in rice (**Figure 4A**). If these two QTLs acted together, then the CSSL D1 that carried both QTLs should show increased GL by 1.46 mm (0.45x2+0.28x2) with respect to the receptor parent Nipp. Hence, the GL of D1 should be 8.39 mm (6.93 + 1.46) if *qG-3* and *qGL6* acted jointly on GL. However, the observed GL of D1 was 7.71 mm (i.e., <8.39 mm), suggesting that *qGL3* and *qGL6* acted antagonistically to each other regarding GL in rice (**Figure 4A**). Furthermore, the *t*-test results showed that the GL of D1 was higher than that of S2 that carried only *qGL6* (*P* <.01), and *qGL6* interacted even negatively with *qGL3*. This result suggests that, in the breeding activities related to GL improvement, we can either utilize *qGL3* independently or pyramid *qGL-3* and *qGL-6* to produce longer grains in rice.

Interestingly, using the F2 population, *qGL-7* for GL was detected on the introgressed segment from Chr7, but no QTL for GL was identified *via* the CSSL S3; even the core marker linked to *qGL-7* was fixed within the introgression segment on Chr7 carried by S3 (**Figure 4A**). However, the phenotypic results showed that the introgressed segment carried by S3 (*Null*) exhibited a weak increasing effect on GL (0.03 mm, **Figure 4A**). Such a weak effect caused by *Null* might have resulted from complex relationships of GL, PH, PL, and other agronomic traits (Zhang et al., 2020). In addition, *Null* interacted with *qGL-6* and exhibited a positive epistatic effect of 0.09 mm to GL (**Figure 4A**). When *qGL-3*, *qGL-6*, and *Null* were pyramided, i.e., T1, the GL was the longest (8.17 mm, **Figure 4A**). These results indicated that using the target QTLs or gene(s) for improvement of candidate trait(s) should be done with a strong consideration of the locus or chromosomal regions without target QTLs.

Despite the increasing effect of *qGW-3* on GW, both *qGW-6* and *Null* had negative effects to GW, and the exacerbated negative trends were observed after pyramiding *qGW-3*, *qGW-6*, and *Null* (**Figure 4B**). Phenotyping results showed that the strongest decreasing effects were caused by the epistatic interaction of *qGW-3*, *qGW-6*, and *Null* (T1), then *qGW-6* and *Null* (D2), and then *qGW-3* and *qGW-6* (D1, **Figure 4B**). These results suggest that, if one tended to decrease GW in rice slightly, one could utilize *qGW-6* alone or combine *qGW-3* and *qGW-6* together, which would cause approximately a 4% decrease of GW. To achieve a moderate decrease of GW, the integration of *qGW-6* and *Null* would tend to produce approximately a 7% decrease of GW. Finally, the pyramiding *qGW-3*, *qGW-6*, and *Null* in breeding activities might result in about a 10% decrease of GW in rice. In addition to the improved phenotypic performances of GL and GW, the pyramiding of target QTLs in T1 also caused side effects for better quality of polished grains in T1 (**Figure 5**), implying more potential utilization for yield and quality improvement of rice varieties.

## Conclusion

In the present study, an elite CSSL named Z1357, screened from the progeny derived from crossing of Nipponbare as the receptor and Xihui18 as the donor, was characterized by carrying nine substitution segments with the average length of 4.13 Mb. Thirteen QTLs were detected on nine substitution segments for the seven traits of interest. Results *via* other CSSLs, i.e., S1 to S3, D1, D2, and T1, showed that pyramiding the segments from Chr3 (*qGL-3*, *qPH-3*, and *qGW-3*), Chr6 (*qGL-6*, *qPH-6*, and *qGW-6*), and Chr7 (*qPH-7* and *qTGW-7*) tended to produce increased GL and PH and decreased GW, providing a potential theoretical basis for enhancing grain yield and quality in rice breeding.

## Data availability statement

The original contributions presented in the study are included in the article/**Supplementary Material**. Further inquiries can be directed to the corresponding authors.

## Author contributions

ZM, XD, SX, QC, XM, and MC performed the experiments, ZM and XD drafted this manuscript. FZ and YL designed the experiments, developed genetic populations, and planned the structure of the manuscript. ZY, FZ, and YL participated in the development of Z1357. All authors read and approved the final manuscript.

## Abbreviations

QTL, Quantitative trait locus; SSSL, Single-segment substitution line; CSSLs, Chromosome segment substitution lines; DSSL, Double-segment substitution line; TSSL, Triple-segment substitution line; SSR, Simple sequence repeat; MAS, Marker-assisted selection; PH, Plant height; PL, Panicle length; GL, Grain length; GW, Grain width; TGW, 1000-grain weight; GPP, Grain number per panicle; SPP, Spikelet number per panicle.
